# Complete Genome Sequence of the Polymyxin E (Colistin)-Producing *Paenibacillus* sp. Strain B-LR

**DOI:** 10.1128/MRA.01013-18

**Published:** 2018-12-13

**Authors:** Caroline Salsano, Sandrine Didelot, Fatoumata Tambadou, Thibault Caradec, Valérie Sopéna, Cyrille Barthélémy, Valérie Thiéry, Romain Chevrot

**Affiliations:** a Université de La Rochelle, Laboratoire Littoral Environnement et Sociétés (LIENSs)–UMR 7266–CNRS, La Rochelle, France; b Institut Pasteur de Lille, Lille, France; Loyola University Chicago

## Abstract

*Paenibacillus* bacteria are recovered from varied niches, including human lung, rhizosphere, marine sediments, and hemolymph. Paenibacilli can have plant growth-promoting activities and be antibiotic producers. They can produce exopolysaccharides and enzymes of industrial interest. Illumina and PacBio reads were used to produce a complete genome sequence of the colistin producer *Paenibacillus* sp. strain B-LR.

## Abstract (precis)

*Paenibacillus* bacteria are recovered from varied niches, including human lung, rhizosphere, marine sediments, and hemolymph. Paenibacilli can have plant growth-promoting activities and be antibiotic producers.

## ANNOUNCEMENT

*Paenibacillus* bacteria are well-known producers of antimicrobial compounds, such as peptides, nonvolatile organic compounds, and enzymes (1). Paenibacilli can produce nonribosomal peptide (NRP) antibiotics, such as polymyxins, fusaricidins, and depsipeptides (2, 3). Paenibacillus polymyxa strains are used by pharmaceutical corporations for their bioproduction of polymyxins B and E (colistin), targeting Pseudomonas aeruginosa infections. *Paenibacillus* sp. strain B-LR was isolated from an environmental sample during a screening of antagonistic bacteria with antagonism against P. aeruginosa PA01 (3). Curiously, even though *Paenibacillus* sp. strain B-LR is related to Paenibacillus alvei and not to P. polymyxa, it is also a polymyxin E producer.

Preliminary sequencing work was done using an Illumina MiSeq (2 × 150-bp) platform (Eurofins Genomics, Germany). Genomic DNA was extracted using the NucleoSpin tissue kit (Macherey-Nagel, Germany) on overnight cultures grown at 30°C in Luria-Bertani broth (Sigma, France) (3). A total of 2,829,200 paired-end plus 3,042,337 singleton reads with an average of 103 bp were obtained using a long jumping distance library with an insert size of 8 kbp. Unfortunately, the obtained *de novo* genome assembly presented issues, especially within the gene cluster responsible for the production of the polymyxin E that we had already sequenced (3). The repetitive regions within the nonribosomal peptide synthetase (NRPS) genes can be misassembled when using short-read sequencing. Therefore, a second sequencing run was performed using PacBio RS II (Pacific Biosciences, USA) by Eurofins Genomics, Germany. Genomic DNA was extracted as described for the Illumina sequencing. Quality controls of the DNA were obtained from a Quantus fluorometer (Promega, USA) combined with NanoDrop 2000 measurements (Thermo Fisher Scientific, USA) and low-melt agarose gel electrophoresis. SMRTbell libraries were prepared with Blue Pippin size selection. A total of 121,905 reads with a mean length of 8,255 bp were obtained. With a genome size of 6.7 Mb, PacBio single-molecule real-time (SMRT) sequencing provided approximately 150× coverage. They were *de novo* assembled into a single contig using Hierarchical Genome Assembly Process 4 (HGAP4) (4). The MiSeq reads were mapped onto the PacBio contig using Mira v4.0 with default parameters in order to correct sequence errors (5, 6). The genome sequence was annotated using the Microbial Genome Annotation and Analysis Platform (MAGE) (7). The *Paenibacillus* sp. strain B-LR circular genome is 6,694,765 bp long with a 46.76% GC content and contains 6,129 coding sequences (CDSs), 81 tRNAs, and 30 rRNAs. The sequence was submitted to antiSMASH analysis of secondary metabolite biosynthesis gene clusters (8). Among the 42 clusters identified, 18 were only putative, whereas 25 were assigned to functions. Eight were involved in NRP biosynthesis, whereas four were involved in hybrid polyketides/NRP biosynthesis. The other clusters were involved in the biosynthesis of 6 saccharides, 4 fatty acids, bacteriocin, lasso peptide, and resorcinol. Among the NRPS clusters, the gene cluster responsible for biosynthesis of polymyxin E was recovered and shown to be fully identical to the one we had previously described (3). The other NRPS clusters shared low gene similarity percentages with those found in the antiSMASH database, which could suggest their novelty.

### Data availability.

This complete genome sequence project has been deposited at GenBank/ENA/DDBJ under the accession number LS992241. The raw sequencing reads are deposited at GenBank under the accession number SRR8135148.
